# Perceived effect of warning label on parental food purchasing and drivers of food selection among South African parents–An exploratory study

**DOI:** 10.3389/fpubh.2022.939937

**Published:** 2022-08-05

**Authors:** Makoma Bopape, Lindsey Smith Taillie, Rina Swart

**Affiliations:** ^1^Department of Human Nutrition and Dietetics, Faculty of Health Sciences, University of Limpopo, Polokwane, South Africa; ^2^Faculty of Community and Health Sciences, School of Public Health, University of the Western Cape, Cape Town, South Africa; ^3^Carolina Population Center and Department of Nutrition, Gillings School of Global Public Health, University of North Carolina at Chapel Hill, Chapel Hill, NC, United States; ^4^Department of Dietetics and Nutrition, Faculty of Community and Health Sciences, University of the Western Cape, Cape Town, South Africa

**Keywords:** food purchasing, food selection, unhealthy food, warning label, parental

## Abstract

Household food purchasing decision is a complex process influenced by factors such as marketing, cost, children food preference and parental choices. Most food products targeted toward children are unhealthy and are aggressively marketed to increase desirability among parents and children making healthier food selection even harder. The warning label (WL) is identified as a simple front-of-package labeling format that assist consumers to easily identify unhealthy foods and reduce their purchasing. This was a qualitative study that aimed to investigate the perceived effect of the warning label (WL) on parental food purchasing and drivers of food selection among parents. The study was conducted in a mainly rural part of South Africa, in Limpopo Province. Data were collected from 44 adult participants, all parents with children aged below 16 years selected using the snowball sampling method. Seven focus groups diversified according to age, literacy, income and urbanicity were utilized for data collection. Using a focus group discussion guide, parents were shown images of six products (crisps, soda, juice, biscuits, cereals, and yogurt) superimposed with the WL and questions asked were based on those images. Thematic analysis revealed that although some parents felt undeterred by the WL, some felt they would alter their food purchasing in the presence of the WL. Other parents felt they would reduce the frequency or the amount purchased or completely stop purchasing labeled products for their children. Motives behind perceived behavior modification included children's health being perceived as a priority and labeled products being viewed as unhealthy. Factors such as pressure from children, taste, poor nutrition knowledge and affordability seemed to influence parental food selection. These findings have important policy implications by providing evidence to policymakers that the WL may alter parental food purchasing and also provide insight into drivers of food selection among South African parents.

## Introduction

Non-communicable diseases account for more than 51% of all deaths in South Africa (1) and are ranked among the top ten leading causes of mortality in the country (2). The link between poor diets and NCDs necessitates public efforts aimed at modifying food purchasing and consumption to reduce the burden of NCDs in the country.

Unhealthy diets are one of the major modifiable risk factors responsible for NCDs (3, 4). Currently NCDs account for more than 85% of premature deaths per year in low- and middle-income countries (5) posing a substantial burden on the economy (3, 6). In South Africa, it is estimated that for diabetes alone, in 2018, the public sector costs of diagnosed patients was approximately R2.7 billion (approximately 157 million USD) and would be R21.8 billion (approximately 1.25 billion USD) if both diagnosed and undiagnosed patients are considered (7).

Although mostly experienced later in life evidence suggests that diet-related NCDs start early in childhood and adolescence (8) Childhood presents a golden opportunity for NCD prevention as any healthy behaviors developed at this stage may have positive long lasting health implications (8, 9). Policies aimed at improving healthy food selection from an early age are seen as cost-effective public measures (9).

Parents are primary household food purchasers and although influenced by other external factors such as time constraints (10), pressure from children (11), taste (12), marketing (13), and food prices (10, 13), they are to some degree responsible for selecting food for their children (13, 14). Parental food selection plays a role in shaping children's health (15) and preventing current and potentially future diet- related-diseases (15, 16). Studies report that parents often base their purchasing decisions on perceived product healthfulness, health claims and attractive packaging (13, 17) rather than on the nutritional value of products (17). Most products targeted toward children are high in nutrients associated with NCDs - energy, fats, salt and/or sugar (18) and are aggressively marketed to increase desirability among children and parents alike (17). In an effort to provide the best for their children, parents are often misled by the attractive packaging and health claims that appear on product packaging (17, 19).

International organizations recommend provision of nutritional information as a strategy to assist consumers identify healthier food options (20, 21). Evidence from previous studies shows that consumers understand and prefer interpretive front-of-pack labeling (FOPL) (22, 23) as it presents nutrition information in a simplified format (21, 24). Interpretive FOPL simplifies nutrition information by providing interpretation or judgement about the nutritional value of products and may appear in the form of color coding, words, pictorial images or symbols (21, 25).

Existing research reveals that in the presence of the WL, an example of an interpretive FOPL, consumers are better able to understand the nutrient quality of food and select healthier food options (23, 26). WL interprets and simplifies nutrition information by presenting it in a form of familiar shapes such as the octagon shape resembling stop signs (27), triangles (28) and some include icons or symbols that represent nutrients that are in excess (21, 29). This is in contrast to the traditional list of nutrition information typically stated at the back of the pack or the non-interpretive FOPL which still require further interpretation by consumers (25). These positive effects of interpretive FOPL on food selection may play an important role in reducing NCDs (30).

**Figure 1:**
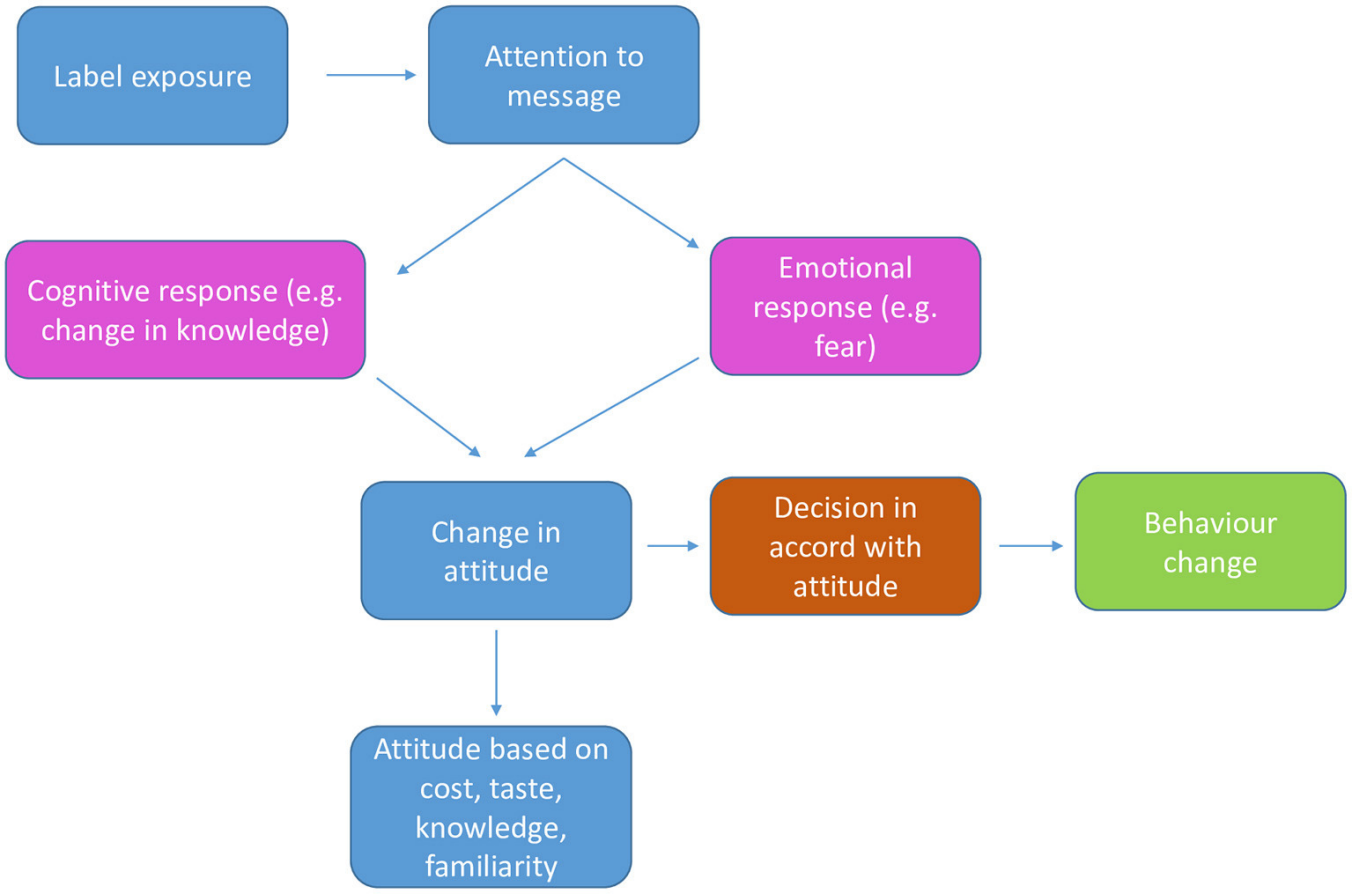
Conceptual framework that explains influence of warning label on food purchasing behavior.

Two conceptual framework models were adapted to explain pathways through which the WL influences purchasing decisions and to secondly explain drivers of food selection (Figure 1) (31, 32). According to the authors, the label needs to first capture consumer's attention. Once attended to, the label can work through two different pathways. The first pathway is through cognitive effects such as improving understanding and subsequently changing product perceptions (31). For example, the WL might assist consumers to understand that a product previously perceived as healthy is in fact unhealthy (23). This nutrition information should be presented in a manner that challenges existing beliefs and attitudes (31, 33) and such labels are likely to have the greatest impact (34). The second mechanism is through eliciting negative emotional reactions such as fear and worry or increasing risk perception (31, 32). In two separate experimental studies, exposure to the WL was reported to elicit negative emotions toward sugar sweetened beverages (35, 36). According to the Health Belief Model, high-risk perception motivates change in beliefs and attitudes and ultimately illicit desired reaction (37). Labels can also influence behavior by simply serving as a salient reminder of one's long-term health goals (31) or reinforcing current health beliefs and attitude (33). These cognitive and emotional influences can in turn affect attitudes toward foods or directly influence behavioral intentions with subsequent changed behavior (26, 38). The effectiveness of the label can however be influenced by income (39) taste (39), cost (40), product familiarity (41) and nutrition knowledge (42).

Warning labels have been reported to positively impact consumer behavior by shifting the desire away from unhealthy products (23, 43). Another study revealed that parents were reluctant to purchase unhealthy products for their children after exposure to the WL (26). In another separate study the application of the WL on products also led to decreased intention to consume and purchase labeled products (44).

A previous study evaluating the opinion of South African adult consumers on WLs revealed that consumers found the labels attention-grabbing, easy to understand and effective in warning against unhealthy food (28). Findings of the latter study revealed that some consumers felt they would reduce consumption of products bearing WLs.

The latter study did not however investigate the perceived effect of the WL on parental food purchasing. Parents seem to select food differently for their children (12, 17) and parental view on the effect of the WL on food purchasing for their children is therefore important. Parental food selection shapes children dietary habits making it crucial to develop policies to guide parental food selection. Investigating drivers of parental food selection provides insights into reasons why parents provide certain foods for their children and forms basis for effective parental nutrition education programs. There is a gap in studies related to the parental determinants of food selection from predominantly rural areas of South Africa. This study aims to fill these gaps by investigating the perceived influence of the WL on parental food purchases, motives underlying these perceptions and drivers of food choices by parents.

## Materials and methods

### Participants

We collected data from seven focus groups consisting of 44 participants residing in Limpopo Province, South Africa. All participants were parents with children below the age of 16 years. Focus groups varied according to age (18–29 years and 30–50 years), income (low and middle-high), literacy level (low literacy and literate) and urban or rural residency (Table 1).

**Table 1:** Socio-demographic characteristics of parents *(n* = *44)*.

	***n* (%)**
**Gender**	
Male	5 (11)
Female	39 (89)
**Age**	
18–29 years	10 (23)
30–50 years	34 (77)
**Urbanicity**	
Urban	18 (41)
Rural	26 (59)
**Literacy**	
Low literacy (grades 0–6)	14 (32)
Literate (grade 7 and above)	30 (68)
**Combined family monthly income**	
Low (R0–R1,600)	33 (77)
Middle-high (R1,601 and above)	11 (23)

Low income was defined as an income below or equal to R1600.00 (approximately 94 USD) and income above R1600.00 was categorized as middle-high. Low literacy was defined as educational attainment at or below Grade 6 and a participant with Grade 7 and above was considered literate. The purpose of diversifying the groups was to capture potential differences in perceptions according to different ages, educational and socioeconomic status and urbanicity. The sample consisted of parents primarily responsible for either purchasing or preparing food within the households and having children below the age of 16 years. MB, one of the researchers, recruited participants both face-to-face and telephonically through the snowball sampling method. Ethical approval was obtained from Biomedical Research Ethics Committee of the University of the Western Cape. The materials and methods followed in this study are presented according to the Consolidated Criteria for Reporting Qualitative Research (COREQ) (45).

### Stimuli

**Figure 2:**
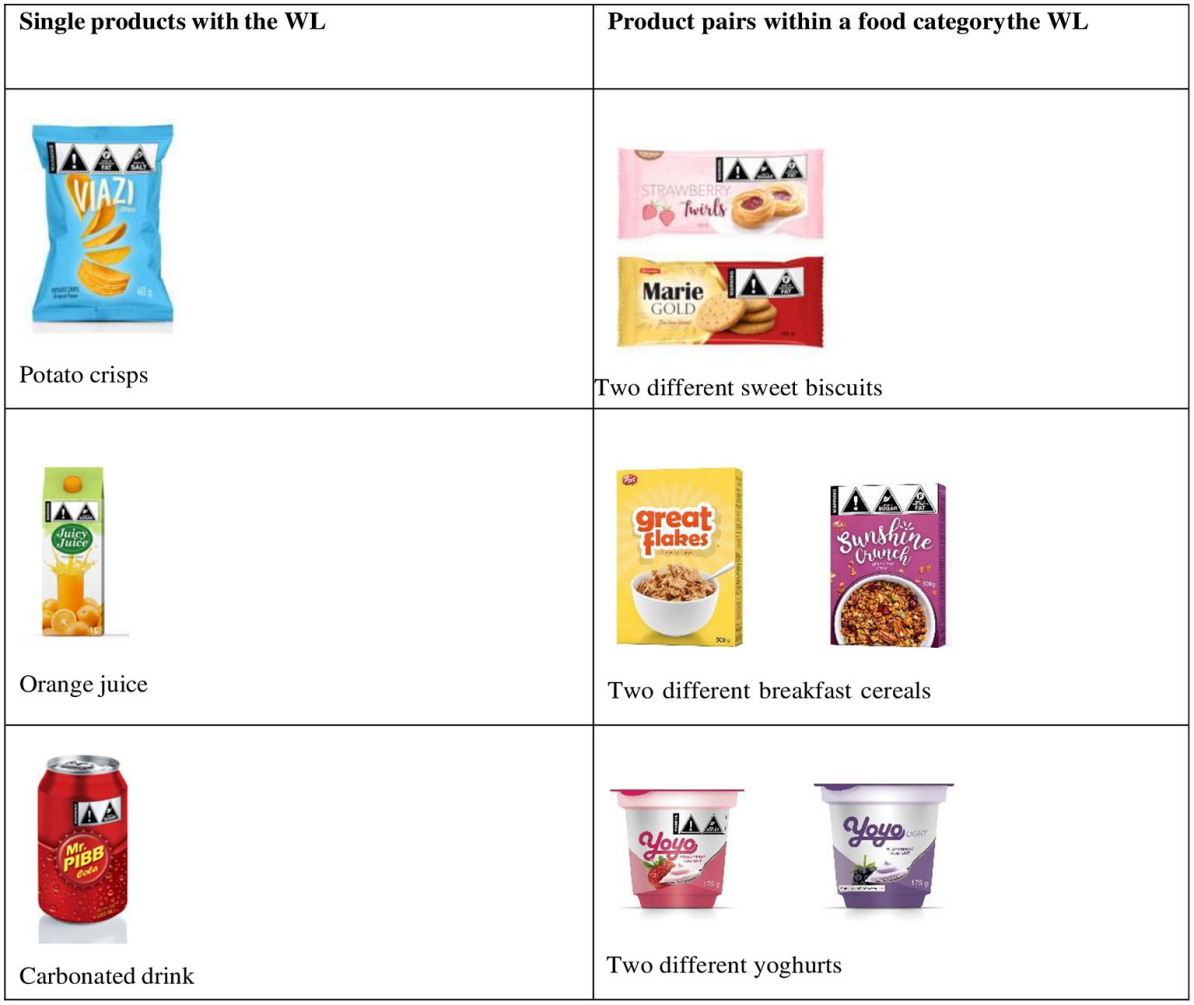
Images used during data collection.

Discussions were based on 2D images of mock-up products (crisps, soda, juice, biscuits, cereals, yogurt) superimposed with the WL (referred to as labeled products in this study) (Figure 2). The nutrient content of each product mimicked a similar product that is currently on the market and each product package contained a WL based on the nutrients that were in excess. For example, a product high in sugar and saturated fats would contain a WL with two triangles indicating “high in sugar” and “high in saturated fats” (Figure 2).

### Procedure

All discussions were conducted by MB using a focus group discussion guide (Additional File 1) developed by the researchers. Data collection took place between November 2020 and December 2020; in March 2021 and in November 2021. The break-up in data collection was due to coronavirus disease 2019 (COVID-19) restrictions. Venues most convenient to the participants were arranged and COVID-19 protocols were observed at all times. Participants kept a safe distance from each other, wore masks all the time and sanitized their hands before discussions started. All focus group discussions were captured on the audio recorder.

Before the commencement of the study, the moderator explained the aim of the study which was to explore the views of the participants on the images to be displayed during discussions. Once the purpose of the study was explained, participants were then requested to sign the focus group confidentiality binding form. Participants were shown different images and responded to questions based on those images. The images were first rotated within the focus group to ensure each participant had a closer view of the images together with all the graphics. Participants were requested to view the images in silence. Once all participants had viewed the images, the moderator presented the images again, one at a time, without providing any explanation, to ensure all participants were aware of all the images. Once completed, the moderator started the discussions with one image, chosen at random, and led the discussions until all responses were pointing to the warning label and not the product. Once participants' focus was on the warning label, the moderator then continued to ask questions based on the focus group discussion guide. Focus group discussions lasted between 40 min to 45 min and were conducted until data saturation was reached was for the questions. Discussions were held in Sepedi, the language that participants understood. MB moderated the discussions transcribed the recordings verbatim and translated the data to English.

### Focus group discussion guide

The researchers developed a focus group discussion guide that was used during focus group discussions. The guide was based on the adapted conceptual framework (31, 32) (Figure 1) which suggest a hierarchy of events that determine the effect of the WL on food purchasing and drivers of food selection. The questions in the guide were aimed at investigating whether the WL caught participants' attention and understood the message conveyed by the WL. The aim of these questions was to ensure that all participants were aware of the WL before the discussions on the perceived influence could commence. Other questions on the discussion guide included the perceived effect of the WL on food choices for their children. Furthermore participants were asked about their impression about the label and for drivers of food choices, participants were asked about other factors that they could consider when making food choices for their children. A previous qualitative study reported that consumers in South Africa found the WL attention grabbing (28) and this paper therefore excludes discussions on this first step of the conceptual framework (Figure 1).

### Data analysis

Data were analyzed following inductive thematic analysis (46). Although the framework was developed beforehand, the researchers allowed codes and themes to emerge from participants responses and not from predetermined codes (46). To ensure robustness of data analysis. MB and another experienced independent researcher (FP) separately analyzed all the transcripts following the iterative process (47) and each grouped similar information into codes. MB and FB discussed the codes and after reaching consensus on codes to include or exclude based on the conceptual framework and any other emerging data related to the framework, we each sorted and collated codes into themes that best represented participants' responses. We compared and finalized the themes based on themes that were common between the two coders. The themes were supported with relevant quotes from participants for further clarity and explanation.

Trustworthiness of the study was ensured throughout the study process (46, 48). To ensure credibility MB built a rapport with participants by stating the purpose of the study and reasons why the researcher was interested in their views (49, 50), starting the discussions with light topics for icebreaking and listening attentively during discussions (49, 50). To ensure confirmability two independent researchers followed similar data analysis steps separately to generate codes and themes and agreed on themes that best represented participants' responses. The authors of this article also reviewed the themes and the quotations. To ensure transferability and dependability the study methodology followed in this study is fully described.

## Results

We extracted six themes with several subthemes from the data.

### Perceived meaning and usefulness of the WL

During discussions all parents were requested to share their views about the WL and the responses ranged from: ***WL cautions against nutrients in excess, WL promotes informed*** ***food choices*** and ***WL reminds of health consequences***.

#### WL cautions against nutrients in excess

A number of parents' remarked that the WL alerts them to nutrients that are contained in excessive amounts, to which one parent said: “*There is too much fat and too much salt in that package” (Male, urban, literate, middle-high income)*. Another parent said*: “And this is high in salt. This tells you that this product contains too much salt” (Female, rural low literacy, low income)*. This implies that some parents were able to correctly understand the message conveyed by the WL.

#### WL promotes informed food choices

One other view from some of the parents was that the WL would enable them to make informed food choices**. **This is what one parent said: “*As a parent, I will be the one going to the shops then I will know which products to buy for my child. I will first check the label and then know how my child will be eating. I will be aware of what I am feeding him (Female, rural, low literacy, low income)”*. Another parent added: “So *if we have inherited diseases in the family and we are diagnosed with certain diseases or hypertension in the family, I*'*m going to check the label first. If the label says this product contains too much fat or too much sugar I*'*m going to stop buying it (Male, urban, literate, middle-high income).”*

#### Warning label reminds of health consequences

From the discussions it was evident that the presence of the WL made a number of parents think about the health consequences related to overconsumption of the labeled products. “*But if you think carefully, you will remember that eating too much sugar and too much salt causes diseases and then you will not buy them” (Female, urban, literate, low income). “I cannot buy a product that will make me sick at the end (Female, rural, low literacy, low income).”*

### Emotional responses to warning labels

When asked how they would react if the WL was implemented and put on products in the supermarkets, the perceived emotional reaction from several parents was fear as illustrated by these responses: “*It will scare us” (Female, rural, literate, low income)*. Another parent in the same group added: “*We will no longer buy as usual, we will start to be afraid” (Female, rural, literate, low income)*. In response to the question, another parent said: “*I will be scared”* (Female, urban, literate, middle-high income) s*:*

### Perceived effect of WL on parental food purchases

Parents were asked about their perceived reaction if products they usually purchased for their children would contain a WL. The following subthemes emerged: ***reduce the amount*** ***and frequency of purchasing, stop buying labeled products, continue buying labeled*** ***products*** and **s*****witch to a different product***.

It was evident that the WL affected a number of parents as noted by quotes such as: “*It*'*s not going to be easy to buy products with labels for them” (Female, urban, literate, low income)* ' indicating some discomfort in buying products should they contain the WL in future. One parent said*: “This label is going to be helpful as we will be able to see that we actually were not feeding our children well and things would have to change (Female, rural). Even myself that*'*s what I normally buy for my kids. So starting today I*'*m going to start paying attention to what I buy for them (Male, urban, literate, middle-high income)*.

#### Reduce the amount and frequency of purchasing and consumption

Although some parents felt they would continue buying labeled products, others felt they would reduce the amount and frequency of buying and consuming such products. One parent said: “*Because mostly what we saw in those pictures is what we normally put in their (children) lunchboxes, which means we are going to cut*' *(Male, urban, literate, middle-high income).”* Another parent said: “*Let me give an example, like with crisps, there are those that come in strips of seven individual packets. I will buy one strip of seven small packets for my child and a big packet for myself, not for my child” (Female, rural)*. Similarly another parent offered*: “Firstly maybe I*'*m going to buy the smaller amount of the pack. So I*'*m not going to buy the big bag” (Male, urban, literate, middle-high income)*. This implied that parents viewed smaller packets of labeled products as better than the big ones.

#### Stop buying labeled products

When asked how the labels would affect food selection for their children, one parent said: “*Ya from my side I will completely stop. I*'*m not going to compromise the life of my children because of the nice time for only a short term*” *(Male, urban, literate, middle-high income)*. Another parent added: “*I will not buy them for my children. We also want them to grow well. If we do not want fat and salt for ourselves, we also do not want it for them” (Female, rural, low literacy, low income)*.

#### Continue buying it

Some parents acknowledged they would continue purchasing labeled products especially when shopping with their children. “*I will continue buying products with labels” (Female, urban, literate, low income)*. Another parent added: “*Personally, I will buy the one with a warning label. A child will not eat one without the label as it will not be containing sugar” (Female, rural, literate, low income)*. This implied that parents were willing to accommodate their children's food preferences.

#### Switch to a different product

Other parents' opinion was that they would instead consider other alternatives to labeled products. *One* parents said: “*I will check for other products without the warning signs” (Female, urban, literate, low income)*. When asked what they would pack for children's lunch if they stopped buying labeled products parents in the current study said they would pack non-labeled products, fruit and water. One parent said: “*We will pack the one without the warning label”* (*Female, rural, low literacy, low income)*. Another parent said this in response: “*Fruit, they are the healthy option. They do not have any negative consequences”* (*Female, rural, low literacy, low income*). When asked what would happen if the child does not enjoy the non-labeled product one parent said: “*They will get used to it”* (*Female, rural, literate, low income*).

### Motivation for modifying purchasing behavior

Some parents further offered their reasons for intention to modify purchasing behavior that included: ***health as a priority*** and ***labeled products viewed as unhealthy***.

#### Health as a priority

A number of parents alluded to the importance of children being healthy from a young age: “Because children should also be healthy from childhood. We should take care of them while they are still small and not only start giving them healthy food when they are older. He should get used to them from an early childhood” (Female, urban, literate, low income).

When asked what they would do should unlabelled products be more expensive, some parents maintained that they would still not buy labeled products for their children. One parent remarked*: “My child*'*s health is a priority. My child*'*s health cannot be compared to any amount” (Female, urban, literate, low income)*.

#### Labeled products viewed as unhealthy

Parents viewed products containing high amounts of nutrients of concern as unhealthy and that seemed to serve as motivation for intention to modify purchasing behavior. There was a general concern about the poor nutritional value of the labeled products. One parent said: “Food high in fat is unhealthy. They can cause diseases” (Female, urban, literate, low income). In addition, another parent said: as you can see, crisps contain salt and; fat is also written there (pointing at the pack). in the body they will just create a mess. We need to be selective with the type of snacks we eat, (choose) healthy ones (Female, rural, low literacy, low income).

### Drivers of parental food selection

During discussions, drivers of food selection mentioned by parents included: ***pressure*** ***exerted by children, taste, poor nutrition knowledge and affordability***. Regarding ***pressure*** ***exerted by children*** one parent said: “*Most of the time we go with them and it*'*s not easy. Because that kid will scream in the shop like you stole him whereas he is yours. even if you agree at home that you are not going to cry for these and that. (Male, urban, literate, middle-high income).”* Another parent felt ***taste*** play a role*: “A child will not eat one without the label (Female, rural, literate, low income).”* Another parent said: “*Lite ones do not taste nice. Children are controlled by sugary stuff, even us. It will stay in the fridge for a long time” (Female, urban, literate, middle-high income)*.

***Poor nutrition knowledge*** also emerged as one of the reasons for food selection. One of the parents believed that children are not at health risks due to their young age: “*I don*'*t think these will affect children that much. Because they are still young they might not get very sick (Female, rural, literate, low income). On the other hand other parents viewed food high in sugar as harmless.” “I don*'*t think there is anything wrong with food high in sugar. For example if cereals are high in sugar, I can eat them with milk and then add no additional sugar” (Female, rural, low literacy, low income). Another parent said: “It is not possible to drink a hot drink (referring to fizzy drink). But you will not even feel its sweetness when it is cold, you just drink, no problem at all” (Female, rural, low literacy, low income). This implied there were compensatory measures one could take to balance the amount of sugar in food*.

Other parents felt compelled to purchase certain foods due to ***affordability**. One* parent said: “*I will buy labeled products if unlabelled products are more expensive” (Female, urban, literate, low income)*. Another one said*: “We give them whatever is available. If it*'*s a month where you managed to buy cheaper ones that*'*s what they will take to school, if you managed to buy expensive ones, that*'*s what they will carry” (Female, urban, literate, middle-high income)*.

When asked how frequently they felt labeled products should be consumed, their responses ranged from one to three times per week. One parent said: “*Once a week — because some of the products are needed in our bodies, even salt must not be a lot, but it is needed. Even sugar and alcohol. Not too much alcohol but just a little bit. We have to balance it like that” (Male, urban, literate, middle-high income)*. Another parent said: “*So we still need snacks but not too often or every day”* and when asked how often this parent said*: “Maybe three times a week” (Female, rural, low literacy, low income)*.

### Perceived WL label understanding among children

As a possible determinant of food selection, parents were asked whether they thought children would understand the WL or not. Parents were divided, with some believing the label would be confusing for children. ***One*** parent said: “*It*'*s only adults and literate people who will be able to read the label. Children and the illiterate will not be able to read it” (Female, rural, low literacy, low income)*. Another parent added*: “They cannot understand what is happening there” (Male, urban, literate, middle-high income). Right, it will take time because they will not understand. Children are just a children, they will not understand what is happening. But in future, that thing will build up (Male, urban, literate, middle-high income)*.

***Strategies to maximize WL effectiveness**:* Following were parents' recommendations to improve awareness of the WL: ***parental education of children at home, education of*** ***children at school*** and ***education through mass media***.

Regarding ***parental education of children at home one*** parent said*: “But the best thing it will need us as parents or whoever is staying with the child to educate them” (Male, urban, literate, middle-high income)*. Another parent held a similar view: *Like if she buys the product and comes home with it, we can inform her that she can eat it but it has negative consequences*' *(Female, urban, literate, middle-high income)*.

Other parents also emphasized the importance of modeling healthy eating habits at home. One parent said: “*Because if I keep on buying, my children will think these products are okay. If these products are not available at home, sometimes they might not have money to buy them”* (*Female, rural, low literacy, low income*).

On the other hand some parents were of the opinion that **e*****ducation of children by*** ***teachers at school*** would be better. Their view was that children were more receptive to teachers than their parents. One parent said: **“***When you tell children not to buy biscuits or other stuff they sometimes think that you just don*'*t want them to eat biscuits, but if they are taught about the label at school…. Sometimes children become moody when you tell them to do school work, but when they are at school they listen and do what teachers instruct them to do. So, if schools could be the ones promoting the label, teach them that such products are not good, they do this to the body… they would be hearing that in their classrooms and they listen to their teachers” (Female, rural, low literacy, low income)*.

Another strategy that emerged during discussions is ***education through mass media***. Parents recommended several avenues such as health education at the clinics, broadcasting over the radio and TV and address by the Ministry of Health. Some parents likened the implementation of the WL with the introduction of face masks for prevention of the spread of the Corona Virus. One parent said: “*But the issue about whether children would understand the label or not, if this could be addressed nationally, by the Minister of Health for example, and it*'*s broadcasted live, same as when the president warns us to be careful, it will not be difficult, just like with masks, we are used to then now. It will not be difficult” (Female, rural, low literacy, low income)*.

## Discussion

This study revealed that a number of parents felt the WL would discourage selection of labeled products for their children. Motives for perceived behavior modification were child health being viewed as a priority and labeled products being viewed as unhealthy. In addition the current study revealed diverse drivers of food selection that included pressure exerted by children, taste, poor nutrition knowledge and affordability.

Some parents in the current study felt the WL enabled them to identify products that were high in nutrients of concern. This finding is supported by other experimental studies where the WL performed better in assisting consumers identify products with high amounts of risk nutrients (51, 52). The WL simplifies nutrition information by explicitly stating nutrients that are contained in high amounts. The inclusion of a triangle shape that is associated with danger (53) and icons related to nutrients of interest (e.g., a heap full teaspoon of sugar) could have also enhanced consumers understanding of the WL, thus increasing its effectiveness (53). Labels that are explicit and improve nutrient understanding are more effective in influencing behavior change (30) and may lead to reduced NCDs.

The WL seemed to have made a number of parents think about the negative health effects of indulging in products bearing the WL. Some parents indicated that the presence of the WL would trigger feelings of fear toward products bearing the label. Similar reactions of fear evoked by the WL and thinking about health harms have previously been reported (54). WLs flag unhealthful products and may raise consumers' awareness about the negative health consequences associated with their overconsumption. According to the Health Belief Model, labels that increase the perception of risk are more effective in altering attitudes and may ultimately result in behavior change (37). Modifying purchasing behavior would go a long way toward reducing accessibility of unhealthy food in the homes and potentially into the communities, contributing to reduced consumption of unhealthy food and resultant lowered NCDs and obesity prevalence.

Regarding the perceived effect of the WL, although some parents felt they would continue purchasing labeled products, should the WL be implemented, others felt they would alter their purchasing behavior. A number of parents felt they would reduce the amount and frequency of purchasing labeled products, others felt they would stop purchasing labeled products while some planned to switch to other alternatives. One of the goals of the WL is to discourage purchasing of unhealthy products (55) and parents in the current study expressed their intentions that align with this objective. A study in the UK reported that parents' intention to purchase sugar sweetened beverages was also reduced post exposure to the WL (35). Other researchers have also reported perceived changes in purchasing behavior post exposure to WLs. For example an experimental study in Colombia revealed that the WL reduced the likelihood to purchase “high in” products as compared to other front-of-package labels (56). Similarly other experimental studies reported reduced intention to purchase products bearing the WL (43, 44).

Parents could imagine using the WLs to help guide purchases for children's lunches. This remark was made upon the realization that products shown during the discussions were those they usually include in their children's lunchboxes. Nathan et al. (57) found that children mostly carried food that were not in line with dietary guidelines and that the majority of children carried discretionary food such as chips and sugar-sweetened beverages in their lunchboxes. Parents in the current study acknowledged the importance of healthier food selection for their children, a positive step in the direction toward behavioral change (31). There is evidence of cardiovascular diseases developing from a young age (58) and parents need to start selecting food wisely in order to inculcate healthy dietary patterns in their children much earlier in their lives.

Enablers of the WL in this study included health being viewed as a priority and labeled products being viewed as unhealthy. Health and nutrition were also previously reported as motivators for parental food choices (12). Parents in this study mentioned diverse factors that drove their purchasing behavior. Some parents in the current study reported succumbing to pressure exerted by children while shopping. This is a concept known as pester power which refers to the ability of children to nag their parents into purchasing products they would have otherwise not bought (11, 59). The products mostly demanded by children are usually high in sugar and fat and are hugely marketed toward children and the adolescents (11). While occasional consumption of unhealthy food is by itself not a health risk, a study in Australia revealed that the more parents gave in to children's food preferences, the lesser the preference for fruit, vegetables and untried foods (12). Pester power has also been associated with overweight and obesity in children and the adolescents (11). Parents in other studies similarly reported compromising healthy food purchasing to accommodate their children's food preferences and demands (10, 24).

Poor nutrition knowledge surfaced as one of the influencers of parental food selection. One view held by parents was that sugar does not pose any health problems for younger children. Others felt that sweetened beverages if taken cold would not have any health repercussions as they lose their sweetness when chilled. Such misconceptions could potentially fuel excess sweetened beverage consumption and obesity among children. Any implementation of WL regulations should therefore be linked to strong health education campaigns to improve label understanding and broader nutrition knowledge. This calls for the need to strengthen nutrition literacy initiatives by the health sector, academia and other non-governmental organizations.

Affordability was mentioned as another factor generally affecting parental food selection similar to findings in other studies (13, 24). Low socioeconomic groups often resort to cheaper and unhealthier alternatives which are typically energy-dense and high in nutrients associated with NCDs (60). Therefore, strategies addressing obesity cannot be isolated from food insecurity issues and regulatory measures when implemented need to ensure that healthy and affordable alternatives are accessible to all population groups (3). However, not all parents were willing to compromise on healthy food on account of food prices in the current study. Similarly another study reported that parents were willing to spend more on healthy food for the sake of their children's health (10).

Regarding parental perception on label understanding, a number of parents felt the WL would be meaningless to children. Parents recommended strategies to improve its effectiveness and that included education of children at home, education of children at school, education through mass media and demonstration of healthy eating habits at home. Similar strategies have previously been recommended (61, 62) and have yielded positive results in other countries. In Chile for example, schools assisted in promoting the WL understanding which led to children encouraging their mothers to purchase fewer labeled products (63).

The strength of this study is that discussions were based on a variety of products commonly classified as unhealthy (e.g., biscuits and soda) and those usually perceived as healthy (e.g., yogurt and muesli). Another strength was that all parents had children below the age of 16 years and were suitable to give views as parents. Understanding parental view on the effect of the WL is an important policy consideration as parents play an important role in shaping children's eating habits. The limitation of this study is that data were collected in only one Province and the results may not be generalizable to the entire population. However, the focus groups were diversified to capture opinions from diverse groups to improve the richness of the data. Another limitation inherent in qualitative studies is that focus group discussions can be easily swayed by one vocal group member. Quantitative studies with a representative sample size could be conducted to understand the widespread perceptions of parents in South Africa. This study was experimental and may not translate directly into actual purchasing behavior. The actual effect can only be determined once WLs are implemented. A potential bias for this type of study is demand effects. To deal with this effect, participants were invited to participate freely and were informed that there were no correct or incorrect responses. Additionally participants were only informed that the study was about their perceptions of the images (pictures) to be displayed during the discussions.

## Conclusion

Based on our results parents believed they would reduce the quantity and frequency of consuming labeled products, stop purchasing labeled products and switch to non-labeled products. Some parents felt they would continue purchasing labeled products. Motives to switch to non-labeled products included health being a priority and labeled products being perceived as unhealthy. Drivers of food selection included pressure exerted by children, taste, poor knowledge and affordability. This study provides more clarity on factors influencing food selection by parents and how policy efforts may influence purchasing behavior of South African parents. These results strengthen the importance of implementing WLs in South Africa to benefit children health.

## Data availability statement

The raw data supporting the conclusions of this article will be made available by the authors, without undue reservation.

## Ethics statement

The studies involving human participants were reviewed and approved by Biomedical Research Ethics Committee; University of the Western Cape. The patients/participants provided their written informed consent to participate in this study.

## Author contributions

MB, LT, and RS conceptualized the study and contributed to the study design. MB collected and analyzed the data and drafted the original manuscript. RS acquired funding for the study and supervised the project. LT and RS reviewed and edited the manuscript. All authors contributed to the article and approved the submitted version.

## Funding

This study was received from Bloomberg Philanthropies, Subcontract # 5108311 with the University of the Western Cape.

## Conflict of interest

The authors declare that the research was conducted in the absence of any commercial or financial relationships that could be construed as a potential conflict of interest.

## Publisher's note

All claims expressed in this article are solely those of the authors and do not necessarily represent those of their affiliated organizations, or those of the publisher, the editors and the reviewers. Any product that may be evaluated in this article, or claim that may be made by its manufacturer, is not guaranteed or endorsed by the publisher.
