# Supplementary material for: Perceived effect of warning label on parental food purchasing and drivers of food selection among South African parents–An exploratory study
Source: Front Public Health. 2022 Aug 5;10:939937. doi: 10.3389/fpubh.2022.939937 (PMC9388905; doi:10.3389/fpubh.2022.939937)
Supplement: Supplementary file 1 [file Table_1.DOCX]

**PHASE III FOCUS GROUP DISCUSSION GUIDE**

**Data collection material:** 1) All participants will fill in a socio-demographic questionnaire before focus group discussions commence. 2) A4 posters with pictures of five mock-up food products superimposed with a warning label will be shown to the participants. The following products will be used: a package of chips, 100% fruit juice, sweet biscuits, cereal and yoghurt. (The same ones that we used in the RCT study)

**Data collection process**

The information leaflet (soft or hard copy) will be shared with participants and all participants will be required to fill in the focus group confidentiality binding form before data collection starts.

The moderator will introduce herself, give a brief background about herself and then explain the purposes of the study. The participants then introduce themselves to get them comfortable with one another.

The moderator explains that she is going to show them posters with five food packages and requests that they look at the packages carefully and freely share their thoughts (the moderator might need to move around the room to allow participants to see the posters clearly. The moderator explains that they will have a discussion around the products and that all responses are acceptable.

The moderator then shows the participants pictures of packaged food products (one at a time) with a warning label and asks: Tell me…..

1. ***What do you see on this picture?*** *(noticeability of the label)*
2. ***What comes to your mind when you see these pictures?***
3. ***What are your thoughts about this label that appears on these products?*** *(understanding, take away message, implications)*

*Probing questions:*

*Is the label noticeable? What makes it to be noticeable or not noticeable?*

*What information do you think this label is trying to convey? What do these labels mean?*

*How do you interpret the label?*

***Now I am going to show you the images again. I want you to think about whether you would purchase these products for your child***

1. ***If this label appears on food products, how would it influence what you buy or not buy for your child/children?***

*Probing question:*

*What are your thoughts regarding this label in relation to the kind of food you would buy for your child?*

*What effect do you think this label will have on foods you put in a lunchbox for your child?* ***(try to probe for each product separately)***

*Would you consider anything else other than the label when purchasing groceries for the house?*

1. ***What is your final impression about the label and the use of food labels by food manufacturers?***

*Probing questions: What do you like or dislike about this label?*

*Who in your opinion would be able to use or not use this label?*

*Do you think children will understand the labels? Please explain*

*What effect do you think the labels will have on your child? Please explain*

1. **General question**

*What difficulties do you encounter when reading food labels on food packages?*
